# Exploring the Changes in IL‐6 and Related Cytokines in Angiogenesis after Tibial Transverse Transplantation in Diabetic Foot Ulcers

**DOI:** 10.1111/os.14221

**Published:** 2024-09-02

**Authors:** Daofei Xu, Chunxia Bai, Rong Hu, Xiaoya Li, Fudie Guo, Dingwei Zhang, Bo Shi

**Affiliations:** ^1^ Chengdu Medical College Chengdu China; ^2^ Department of Orthopaedics Mianyang Central Hospital Mianyang China

**Keywords:** Angiogenesis, Cytokines, Diabetic Foot Ulcers, Postoperative Inflammation, Transverse Tibial Transfer Technique

## Abstract

**Objective:**

The transverse tibial transfer technique is employed primarily to treat diabetic foot ulcers (DFUs), aiming to enhance leg circulation and promote new blood vessel growth. This technique is also beneficial for various conditions associated with poor blood flow in the lower extremities. However, there is no clear molecular mechanism to explain the relationship between the transverse tibial transfer technique and angiogenesis in patients with diabetic foot. This study aims to preliminarily explore the change of IL‐6 and related cytokines in promoting angiogenesis during transverse tibial transplantation, providing a direction for future research.

**Methods:**

We retrospectively assessed a study from April 2022 to November 2023 on 76 patients with severe DFUs at Wagner stages 3–4. Flow cytometry was used to detect the levels of 12 cytokines in serum before the operation and 3, 7, 14, 21, and 35 days after the operation. Ankle‐brachial index (ABI), transcutaneous oxygen tension (TcPO_2_), and glycosylated hemoglobin (Hba1c) were recorded at admission and discharge. We examined the variations in cytokine levels, wound healing duration, amputation rates, infection incidence, and other key outcomes.

**Results:**

In our investigation, a total of 76 individuals participated, comprising 49 males and 27 females. These subjects had an average age of 64.7 years, with a standard deviation of 13 years. The mean ulcer healing time was 74 ± 31 days, amputation occurred in 3 patients, pin tract infection occurred in one patient (1.3%), and incision infection occurred in one patient (1.3%). By day 35 following the surgery, both the ABI and TcPO_2_ values showed a significant increase from their preoperative levels. HbA1c significantly improved compared with presurgery (*p* < 0.001), IL‐6 levels were significantly increased compared with presurgery (*p* < 0.05), and then decreased.

**Conclusion:**

The transverse tibial transfer (TTT) technique is safe and efficient for managing DFUs. The wound healing time in patients who smoke or consume alcohol is statistically significant compared with that of nonsmoking and nondrinking patients. IL‐6 exhibited substantial changes at various postoperative time points. Future research could investigate the role of IL‐6 in tibial transverse translation.

## Graphical abstract

The transverse tibial transfer (TTT) technique improves healing in DFUs by enhancing circulation and angiogenesis. This study examines changes in IL‐6 and other cytokines about TTT. We analyzed 76 patients with DFU, measuring cytokines before surgery and at several post‐op intervals. IL‐6 levels initially increased but then decreased.

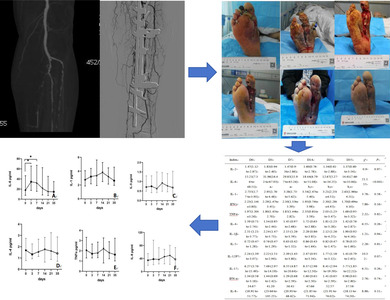

## Introduction

Diabetic foot ulcers (DFUs) are among the most common and severe complications linked to diabetes. Over 15% of DFUs result in lower limb amputations,^1^ and the 5‐year mortality rate is as high as 70%.^2^ DFUs is caused by multiple factors, peripheral artery disease, and microvascular dysfunction have important roles.^3^ Vascular lesions not only cause foot ulcers but also lead to subsequent healing failure and amputation.^4^ Previous treatments for DFUs included surgical debridement and vascular reconstruction. These therapies, due to their complexity, narrow clinical indications, invasiveness, and high cost, have difficulty achieving good clinical outcomes.^5^ Even after expensive treatment and healing of DFU ulcers, the recurrence rates at 1, 3, and 5 years are 40%, 60%, and 65%, respectively.^6^

The tibial transverse transport (TTT) technique is based on the Ilizarov technique. It involves continuous stable adjustment of traction, accompanied by the formation of a rich vascular network. In diabetic foot patients, the area of bone transfer and distal limbs experience extensive neovascularization of capillaries.^7^ Although TTT technology has been proven to improve lower limb blood supply, the mechanism by which it mediates vascular regeneration is not yet clear.^8^, ^9^

Inflammation and angiogenesis are interdependent. Cytokines, with a focus on IL‐6, play a significant role in angiogenesis.^10^ Depending on the actions of cytokines, they can be classified into pro‐inflammatory or anti‐inflammatory categories. Cytokines are essential mediators in the communication network of the immune system, and they play a vital role in immune responses.^11^ They dynamically regulate the growth, maturation, and responsiveness of immune cells, which is crucial for maintaining good health.^12^ Different types of cells secrete individual cytokines, and they can impact various cell types, leading to multiple biological activities.^13^ The fluctuations in cytokine concentrations provide valuable information for the identification, classification, and prediction of various illnesses. Examining changes in cytokine levels aids in investigating their fundamental mechanisms. Our objective is to investigate the influence of TTT on the recovery of patients with DFU, as well as on amputation rates, ABI, TcPO_2_, and associated complications, with the aim of transcutaneous tibial revascularization (TTT) on the recovery of DFU patients, including its effects on amputation rates, ankle‐brachial index (ABI), transcutaneous oxygen pressure (TcPO_2_), and related complications. Additionally, we aim to preliminarily explore the change of IL‐6 and related cytokines in promoting angiogenesis during transverse tibial transplantation, providing a direction for future research.

## Methods

### Patients

Our research focused on diabetic foot patients receiving the same surgical procedure at Mianyang Central Hospital. From April 2022 to November 2023, the same doctor performed all the operations. This consistency in the surgical procedure aimed to minimize variability and ensure the reliability of the study's outcomes. The written informed consent has been obtained from all the patients. This study was approved by the institutional review board of Mianyang Central Hospital (S20230225‐01).

Patients were enrolled if they satisfied the following criteria: (i) patients presenting with diabetic foot ulcers classified as Wagner Grade 3–4 and (ii) all patients underwent transverse tibial transfer.

The exclusion criteria were as follows: (i) ulcers not linked to diabetes; (ii) those with major heart or kidney diseases unfit for surgery; (iii) infections that aren't under control, including those at the site of the incision; (iv) lower leg skin conditions not suitable for surgery; (v) taking medications that may interfere with study results, such as certain anti‐inflammatory or immunosuppressive drugs.

### TTT Technique

**FIGURE 1:**
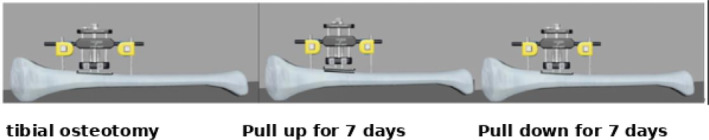
Schematic diagram of adjustment of surgical external fixator.

The tibial transverse transfer (TTT) procedure comprises the following steps: The procedure begins with either epidural or general anesthesia, followed by applying a tourniquet to the mid‐thigh area, maintained for approximately 10 min. The osteotomy site is mapped out on the anteromedial upper‐middle section of the affected tibia, measuring around 20 mm in width and 60 mm in length. In the designated osteotomy area, two longitudinal incisions, each about 15 mm, are made initially. Subsequently, a bone window of 20 × 60 mm is created at the tibia's medial crest using a 2.5‐mm closed osteotome. Two 2.5‐mm diameter holes are drilled into this bone segment, followed by the insertion of two transverse tension pins. Additionally, two 4.5‐mm diameter fixation pins are drilled into both the upper and lower ends of the tibia, parallel to the tension pins. After cutting through the bone cortex with a fine bone saw, the tibial transverse transfer device is installed. Postoperatively, from the 7th day depending on wound recovery, the tibial bone window is gradually moved laterally, ascending 1 mm daily, accomplished in six stages. After a week, this upward movement ceases, and the tibial bone window is maneuvered downward at the same rate, also in six stages. Following another 7 days, the movement is halted, and the tibial bone window is reset. Two weeks later, an X‐ray examination is conducted to check for any complications. If none are found, the tibial transverse transfer device is removed (refer to Figure 1).

### Post‐Operation

Post‐surgical care for patients with DFUs follows established guidelines, initiating with debridement to remove necrotic tissue. When required, the procedure is extended to excise inflamed granulation tissue, as well as any exposed necrotic tendons and bone. Routine cleansing and sterilization of the wound, utilizing iodine and saline, is pivotal in the treatment regimen. Complementary to this is meticulous dietary regulation and insulin treatment, either *via* oral administration or injection, to maintain fasting blood glucose below 8.0 mmol/L. The administration of antibiotics is restricted to instances of evident systemic infection or sepsis. While hospitalized, patients are exposed to a range of therapeutic measures intended to enhance circulatory dynamics, support neuronal integrity, and improve microvascular flow. The schedule for updating ulcer dressings is governed by the exudate level produced by the wound.

### Follow‐Up

To assess the recovery and treatment outcomes of surgical patients, we have implemented a detailed follow‐up method and data collection process. Upon admission, patients undergo comprehensive blood analysis, ABI, TcPO_2_, and other routine preoperative examinations. Serum samples are collected from patients on preoperative and postoperative days 3, 7, 14, 21, and 35, processed, stored at −80°C, and analyzed for cytokine levels (IL‐2, IL‐6, IL‐1, IFNγ, TNFα, IL‐4, IL‐1β, IL‐5, IL‐12P70, IL‐17, IFN‐α, and IL‐8) using flow cytometry. On postoperative day 35, we document TCPO_2_, ABI, laboratory test results, and other perioperative observation indicators until patients' conditions stabilize for discharge. All specimens and data collection follow standardized procedures during hospitalization.

#### ABI Measurement Method

##### Necessary Equipment: Sphygmomanometer

Measurement Conditions: (i) Avoid alcohol, exercise, and caffeine intake within 2 h before measurement. (ii) The patient should be in a supine position, resting for 10 min, with limbs (arms and legs) exposed.

Brachial Artery Systolic Pressure: (i) Wrap the cuff around the upper arm at the level of the heart. (ii) Place the cuff's sensor end over the brachial artery pulse point, 2–3 cm above the elbow crease. (iii) The cuff should be snug enough to insert one finger underneath. Record the brachial artery systolic pressure.

Ankle Artery Systolic Pressure: (i) The measurement site is 2–3 cm above the ankle joint. (ii) Position the cuff's sensor over the posterior tibial artery pulse point, typically on the inner posterior side of the ankle. (iii) Adjust the cuff snugly according to the shape of the patient's calf. (iv) Record the ankle artery systolic pressure.

ABI Calculation: (i) Perform two measurements with an interval of at least 1 min. (ii) Calculate the ABI by averaging the two ankle artery systolic pressures and dividing by the brachial artery systolic pressure.

### Statistical Analysis

The examination of data was carried out with SPSS software, version 26.0. While quantitative data are expressed as mean ± standard deviation, categorical data are illustrated using frequencies and percentages. Comparative analysis of preoperative and postoperative parameters employed paired sample *t*‐tests. For non‐normally distributed datasets, the Wilcoxon rank‐sum test was applied, presenting results as medians (P50) along with interquartile ranges. A *p*‐value < 0.05 was deemed indicative of statistical significance.

## Results

### General Information

This research encompassed a cohort of 76 individuals, consisting of 49 men and 27 women. Regarding the severity of DFUs, the study identified 50 instances as Wagner Stage 3 and 26 as Wagner stage 4. Importantly, a substantial segment of this cohort had preexisting health conditions: 26 participants (34.2%) had hypertension diagnoses, and 37 (48.7%) disclosed having cardiovascular and cerebrovascular histories (Table 1).

**TABLE 1:** General characteristics.

Variables	Total (*n* = 76)
Demographics	
Age (year, Mean ± SD)	64.7 ± 13.0
Male sex, *n* (%)	49 (64.5)
Hypertension, *n* (%)	26 (34.2)
Hypertension duration (years)	8.6 ± 6.7
Smoking, *n* (%)	34 (44.7)
Smoking history (years)	37.9 ± 13.8
Drinking, *n* (%)	31 (40.8)
Drinking history (years)	32.14 ± 13.2
Diabetes duration (years)	11.1 ± 8.5
Well‐controlled blood sugar, *n* (%)	17 (22.4)
Wagner classification, *n* (%)	
III	50 (65.8)
IV	26 (34.2)
Comorbidities, *n* (%)	
Diabetic nephropathy	53 (69.7)
Diabetic eye disease	49 (64.5)
Cardiovascular disease	37 (48.7)
Laboratory test	
Hemoglobin A1c (%)	9.7 ± 2.2
Fasting blood sugar (mmol/L)	11.8 ± 6.0
Postprandial blood sugar (mmol/L)	17.6 ± 6.9
Hemoglobin (g/L)	107 ± 21
Albumin (g/L)	33.7 ± 5.4
Total protein (g/L)	66.0 ± 7.5
Glomerular filtration rate (%)	56.3 ± 19.0
Ankle‐brachial Index (ABI)	0.93 ± 0.22
Transcutaneous Oxygen Partial Pressure(TcPO_2_)	92.7 ± 7.2

During the follow‐up period of the study, a notable majority, 70 out of 76 patients (92.1%), experienced wound healing within an average of 74 ± 31 days. Surgical interventions were required for 3 patients (3.9%), who underwent major amputations. Complications were infrequent, with one patient (1.3%) developing a pin tract infection and another patient (1.3%) encountering an incision infection (Table 2). In the study, the duration of wound healing varied according to the Wagner stage of the DFU. Patients exhibiting Wagner stage III ulcers experienced healing within an average timeframe of 68 ± 27 days, while those with stage IV ulcers required more time, healing in 89 ± 35 days. patients who smoked (*p* = 0.048) and consumed alcohol (*p* = 0.043) tended to need longer periods for wound healing. Additionally, the research indicated no substantial differences in the duration of healing between subjects with a history of cardiovascular and cerebrovascular diseases and those without such histories. Similarly, hypertensive patients had recovery duration that were comparable with those of non‐hypertensive patients (Table 3).

**TABLE 2:** Main outcome measure.

Items	Values (*N* = 76)
Healing (*n*, %)	70 (92.1)
Time to healing (days)	74 ± 31
Amputation (*n*, %)	3 (3.9)
Death (*n*, %)	3 (3.9)
Incision infection (*n*, %)	1 (1.3)
Nail tract infection (*n*, %)	1 (1.3)

**TABLE 3:** Effects of various factors on wound healing time.

Characteristic	Number of patients	Time to heal (days)	*F*	*p*
Hypertension				
Yes	22	70 ± 35	1.237	0.272
No	47	78 ± 30		
Smoking history				
Yes	28	80 ± 37	4.135	0.048
No	41	70 ± 27		
Alcohol consumption				
Yes	26	87 ± 35	4.343	0.043
No	43	66 ± 26		
Cardiovascular disease				
Yes	30	72 ± 31	0.325	0.571
No	39	75 ± 32		
Wagner classification				
III	48	68 ± 27	13.395	0.001
IV	21	89 ± 35		

### Clinical Assessment

**FIGURE 2:**
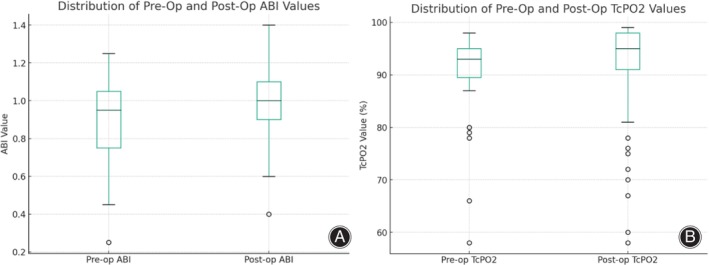
Box plot before and after surgery. (A) ABI. (B) TcPO_2_.

By day 35 following the surgery, both the ABI and TcPO_2_ values showed a significant increase from their preoperative levels (*p* < 0.05) (Figure 2). Additionally, there was a notable decrease in the HbA1c level from 9.6 ± 2.2 preoperatively to 7.2 ± 1.2 postoperatively, marking a statistically significant reduction (*p* < 0.05) (Table 4). Post‐surgery, the improvements in TcPO_2_, ABI, and HbA1c were all significant when compared with their levels before the operation, indicating substantial enhancements in these clinical parameters.

**TABLE 4:** Laboratory indicators of patients.

Index	Pre‐operation		Post‐operation		*t*/*Z*	*p*
	*n*	Mean	*n*	Mean		
Albumin (g/L)	68	33.65 ± 5.50	68	33.53 ± 6.05	−1.934	0.057
Fasting glucose (mmol/L)	58	11.43 ± 5.73	58	11.64 ± 14.80	−0.105	0.917
Glomerular filtration rate (mL/min)	68	56.2 ± 19.6	68	55.3 ± 22.7	1.716	0.092
Hemoglobin A1c (%)	58	9.6 ± 2.2	58	7.2 ± 1.2	8.516	<0.001*
Hemoglobin (g/L)	75	107 ± 21	75	106 ± 21	0.140	0.889
Total protein (g/L)	68	65.86 ± 7.50	68	65.75 ± 9.15	0.093	0.926
ABI	76	0.95 (0.75–1.05)	74	1.0 (0.9–1.1)	−3.172	0.002*
TcPO_2_ (%)	76	93 (90–95)	73	95 (90–98)	−2.417	0.016*

*Note*: ABI and TcPO_2_ values were analyzed using paired sample Wilcoxon tests, while the other indices utilized paired sample *t*‐tests; *p*‐value <0.05 indicates statistical significance when compared with preoperative values.

### Cytokines

**FIGURE 3:**
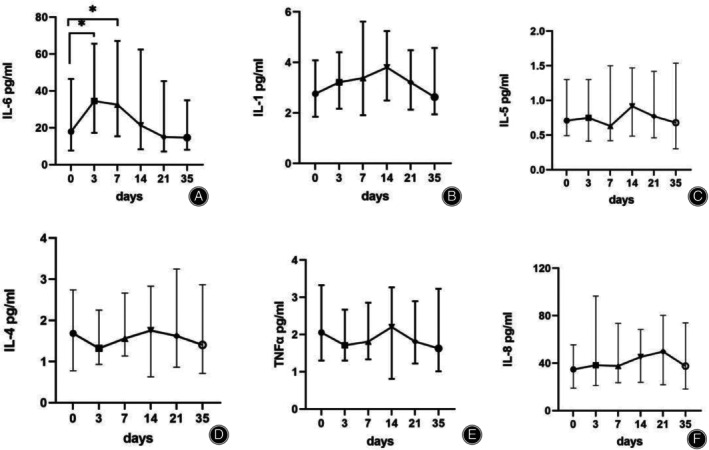
Expression of Cytokines Over Time. (A) IL‐6. (B) IL‐1. (C) IL‐5. (D) IL‐4. (E) TNFα. (F) IL‐8. * indicates *p* < 0.05 compared with preoperative measurements, highlighting statistically significant changes.

**FIGURE 4:**
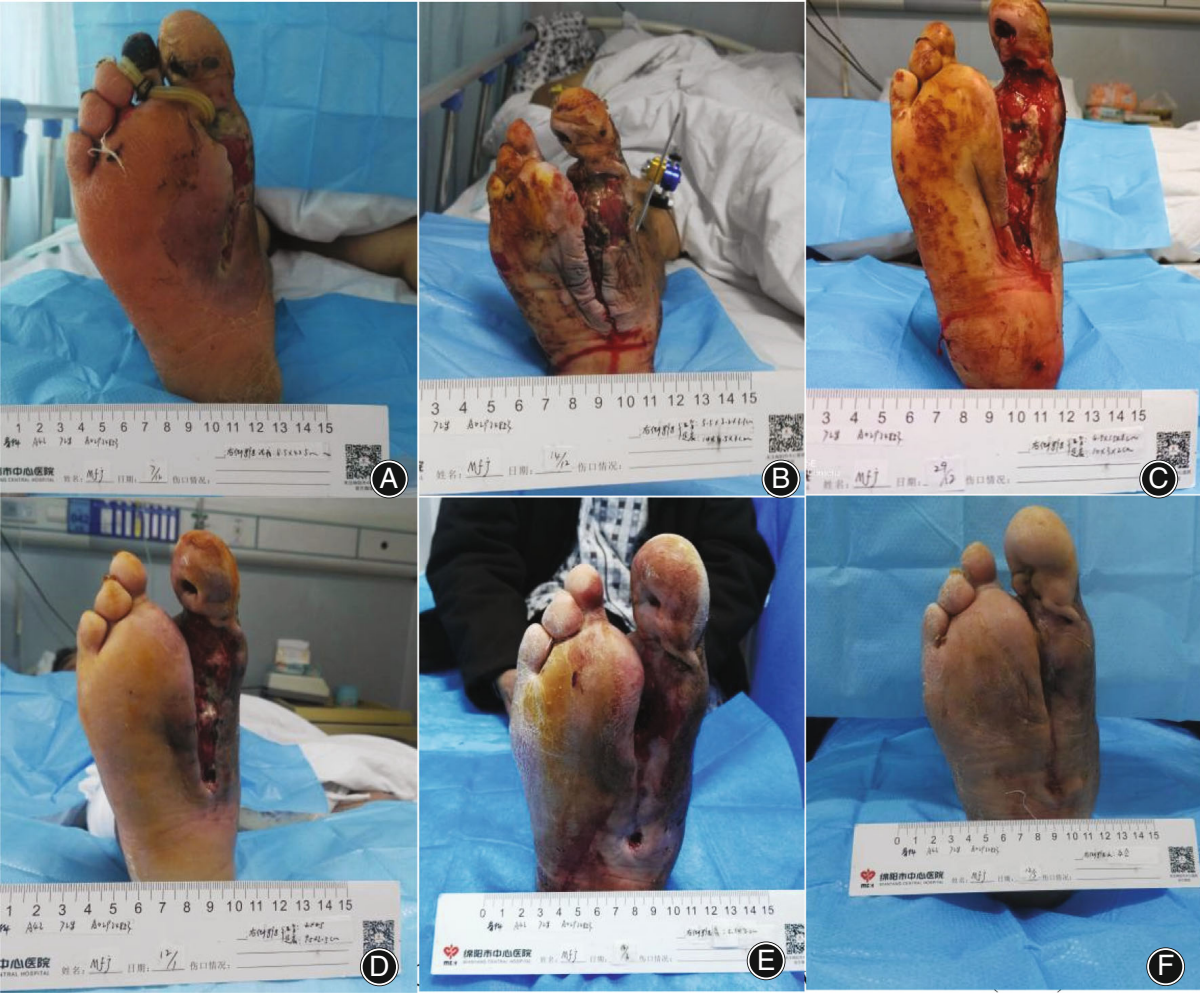
Progression of a typical case treated with Tibial transverse transfer (TTT).

This retrospective study analyzed diabetic foot patients treated with TTT technology at our hospital from April 2022 to November 2023, with an average hospital stay of 39 days. Cytokine testing was conducted at six time points for 52 patients, with 7 patients missing data having one‐time points and 3 having two‐time points. Multiple imputation was used to handle missing data, and the reported data consisted of (*N* = 52) results. In this study, there was a notable rise in IL‐6 expression levels observed on the 3rd and 7th days following the surgery, compared with the levels measured before the operation (*p* < 0.05). Interestingly, the expression levels of IL‐6 on days 14, 21, and 35 post‐surgery were markedly lower than those recorded on the 3rd and 7th days (*p* < 0.05) (Figure 3; Table 5). This pattern suggests an initial upregulation of IL‐6 following the osteotomy, which subsequently returned to baseline levels (Figure 4). Additionally, a similar trend was noted for IL‐1. This was characterized by an initial surge post‐surgery, later returning to the levels seen prior to the surgical procedure (Figure 3).

**TABLE 5:** Expression of coagulation factors in serum (pg/mL).

Index	D0	D3	D7	D14	D21	D35	χ^2^	*p*
IL‐2	1.47 (1.12–2.87)	1.83 (0.94–2.40)	1.47 (0.9 3–2.86)	1.60 (0.76–2.78)	1.34 (0.61–2.88)	1.37 (0.60–3.54)	0.9	0.97
IL‐6	15.21 (7.36–48.51)	31.96 (16.42–67.93)^a^	29.85 (15.97–65.26)^a^	18.44 (6.79–51.08)^b^, ^c^	12.67 (5.27–34.25)^b^, ^c^	14.61 (7.66–33.86)^b^, ^c^	21.155	<0.001
IL‐1	2.735 (1.77–3.93)	2.95 (1.76–4.48)	3.38 (1.73–5.62)	3.54 (2.47–5.40)	3.21 (2.25–4.51)	2.63 (1.96–4.51)	3.78	0.58
IFNγ	2.23 (1.14–3.80)	2.29 (1.47–3.45)	2.30 (1.33–3.39)	1.95 (0.74–3.98)	2.30 (1.28–4.45)	1.70 (0.69–4.10)	7.86	0.16
TNFα	1.97 (1.30–3.26)	1.80 (1.45–2.70)	1.85 (1.44–2.82)	2.35 (0.81–3.59)	2.03 (1.23–3.47)	1.68 (0.95–3.32)	2.22	0.82
IL‐4	1.59 (0.71–2.74)	1.34 (0.85–2.44)	1.45 (0.97–2.68)	1.72 (0.63–2.88)	1.81 (1.23–3.28)	1.42 (0.76–2.87)	6.55	0.26
IL‐1β	2.51 (1.21–3.77)	2.24 (1.47–3.73)	2.15 (1.26–3.59)	2.28 (0.86–3.85)	2.12 (1.26–4.25)	1.90 (0.92–4.16)	1.21	0.94
IL‐5	0.72 (0.47–1.28)	0.76 (0.47–1.29)	0.63 (0.42–1.52)	0.86 (0.43–1.64)	0.82 (0.47–1.47)	0.78 (0.35–1.60)	2.28	0.81
IL‐12P7	2.24 (1.39–3.07)	2.22 (1.51–2.94)	2.39 (1.43–3.80)	2.47 (0.91–3.34)	1.77 (1.18–3.12)	1.41 (0.79–2.65)	10.32	0.07
IL‐17	6.27 (3.76–15.49)	7.48 (2.97–14.19)	8.55 (4.87–20.64)	8.37 (2.98–12.50)	8.41 (2.94–19.59)	5.77 (1.85–22.21)	6.51	0.26
IFN‐α	1.34 (0.58–3.18)	1.44 (0.69–2.42)	1.29 (0.68–2.59)	1.60 (0.61–2.50)	1.41 (0.65–2.59)	0.98 (0.61–2.80)	2.76	0.74
IL‐8	34.67 (18.91–51.77)	41.20 (23.66–105.25)	36.41 (20.93–68.62)	47.66 (21.85–71.94)	52.57 (21.91–76.02)	37.56 (18.11–74.20)	8.88	0.11

^a^ *p* < 0.05 compared with preoperative measurements.

^b^ *p* < 0.05 compared with D3 measurements.

^c^ *p* < 0.05 compared with D7 measurements.

The graphic displayed illustrates the recuperative course for a 72‐year‐old individual diagnosed with type 2 diabetes and hypertension, afflicted with a DFU rated as Wagner stage IV. Each panel captures a different stage of the healing process post‐TTT treatment. It is noted that the ABI and TcPO_2_ values improved to 0.9% and 94%, respectively, 1 month post‐surgery, indicating significant progress in healing. (A) Preoperative appearance of the foot ulcer. (B) Three days post‐surgery, following second toe amputation and extensive drainage, showing a large wound on the sole. (C) Fifteen days post‐surgery, with granulation tissue covering the wound surface. (D) One month post‐surgery, exhibiting more prominent, redder granulation tissue, no signs of necrosis or infection, and beginning of epithelialization at the wound edge. (E) The wound healed basically 3 months after surgery. (F) The wound healed completely 6 months after surgery.

## Discussion

The TTT technique has accumulated extensive surgical experience in clinical practice. During surgeries, strict adherence to preoperative indication assessments ensures detailed preparation of patients, including comprehensive evaluations of soft tissue status, bone quality, and instrument readiness. Intraoperatively, minimally invasive techniques are employed, resulting in smaller incisions. Emphasis is placed on the debridement of necrotic soft tissues without enlarging the wound, aiming to protect soft tissues and achieve effective drainage, thereby significantly reducing soft tissue damage. Key steps such as bone resection methods, size, and location are strictly followed according to predefined protocols to ensure surgical accuracy and safety. Regarding postoperative care, particular emphasis is placed on nail tract care. Utilizing standardized nursing measures, infections and other complications related to the Nail tract infection can be effectively prevented.

TTT technology currently has controversial specific relocation plans, with differing opinions among scholars on the specific operational plans. Whether upward relocation or downward compression affects the vascular regeneration mechanism remains unclear and requires further research. Some scholars suggest pulling upward for 7 days, pausing for 3 days, and then compressing downward, mainly considering incision reasons. However, this study adopts a method of moving 1 millimeter six times a day, considering wound protection and patient tolerance. Therefore, further research is needed in the future to optimize and standardize the operational protocols of the TTT technique, to better guide clinical practice and enhance treatment outcomes.

In our study, we monitored the postoperative cytokines levels in 52 diabetic foot patients. Notably, IL‐6 levels exhibited a significant increase post‐surgery compared with presurgery levels. According to the results, serum IL‐6 reached its maximum value 3 days after surgery and then began to decline. While the peak in IL‐6 levels in the early postoperative period is likely associated with acute inflammatory responses, the subsequent decline and stabilization of IL‐6 levels may also be influenced by the TTT initiated at 7 days post‐surgery. If TTT has reparative or anti‐inflammatory effects, it could potentially accelerate the decrease in IL‐6 levels or stabilize them further. Therefore, the possibility that TTT initiated at 7 days post‐operation affects IL‐6 levels should not be ruled out. Could conduct further research to explore the potential impact of TTT on IL‐6 and other related biomarkers. Extended follow‐up periods and more detailed data analysis methods should be considered to better understand these effects. Furthermore, there was a marked improvement in ABI, TcPO_2_, and HbA1c levels post‐surgery. ABI and TcPO_2_ are commonly used to assess blood circulation,^14^ confirming the TTT technique's crucial role in improving lower limb blood supply and promoting wound healing. HbA1c levels are an important indicator for monitoring and diagnosing diabetes and its control level. This indicates the mean blood glucose levels over the previous 2–3 months.^15^ The improvement in HbA1c levels could be due to enhanced insulin sensitivity and glucose metabolism, resulting from increased blood supply to the lower limbs.^16^ Yan Chen *et al*. conducted a prospective multicenter cohort study to assess postoperative changes in foot arteries and perfusion using computed tomography angiography and perfusion imaging at 12 weeks post‐surgery.^7^ Compared with preoperative measurements, patients exhibited increased numbers of small arteries and higher foot blood flow (8.1 ± 2.2 vs. 28.3 ± 3.9 mL/100 g/min, *p* = 0.003) and volume (1.5 ± 0.3 vs. 2.7 ± 0.4 mL/100 g, *p* = 0.037) at 12 weeks postoperatively. Additionally, pre‐and postoperative coronary CT scans confirmed significant neoangiogenesis in the transplanted area. The ABI, a critical indicator of limb blood supply, showed natural improvement with substantial new vessel formation compared with preoperative levels. Meanwhile, authors such as Xing‐xi Hu *et al*. conducted a systematic review and meta‐analysis using PubMed, Embase, and CENTRAL platforms to evaluate studies on treating DFUs with transcutaneous tibial revascularization (TTT).^17^ Their analysis included seven studies with 818 participants, of whom 661 underwent TTT. Meta‐analytical results demonstrated a significant improvement in ABI (mean difference: 0.23; 95% CI: 0.03–0.44; *p* < 0.001) at the final follow‐up compared with baseline values. This is consistent with the results of this stud. Furthermore, the wound healing time in patients who smoke or consume alcohol is statistically significant compared with that of nonsmoking and nondrinking patients. This suggests the need for a multifaceted management of patients, combining surgery with lifestyle changes to improve the treatment outcomes of diabetic foot.

### The Role of Cytokines in Angiogenesis

Adequate blood supply plays a crucial role in wound healing. Angiography confirmed the formation of numerous new capillaries in the area of bone transfer during the process.^17^, ^18^ Inflammation and angiogenesis are interdependent processes. Cytokines have been found to play a significant role in angiogenesis.^19^ This study found an upregulation of interleukin 1/6 expression after osteotomy, which then returned to baseline levels. IL‐6 has been proven to act as a pro‐angiogenic factor influencing blood vessel formation. For instance, transgenic mice overexpressing IL‐6 exhibited excessive cerebellar vascularization.^20^ Conversely, mice deficient in IL‐6 showed a reduced vascular response to wound injury.^21^ Furthermore, IL‐6 has been observed to induce tumor cells to produce Vascular Endothelial Growth Factor (VEGF), thereby facilitating the formation of blood vessels. IL‐6 is a cytokine that not only promotes inflammation but also plays a vital role in vascular generation.^22^ It fosters blood vessel formation through both VEGF‐dependent and independent pathways. These pathways include the classical activation of macrophages by IL‐6, which indirectly stimulates vascular generation, as well as the interaction of IL‐6 with monocyte‐derived soluble IL‐6R to trans‐activate endothelial cells.^23^, ^24^

Furthermore, our research indicates that variations in cytokine levels, such as IL‐1 and IL‐4 following surgery, point to their significant roles in blood vessel formation. IL‐1, known for its inflammatory properties, is crucial for angiogenesis.^25^ Initially identified as hematopoietin‐1, IL‐1 was named for its angiogenic capabilities.^26^ It operates by interacting with its receptor to initiate downstream signaling pathways. Post‐activation, MyD88 forms an association with interleukin receptor‐associated kinase 4, leading to the activation of downstream MAPK and IKK/NF‐κb signaling pathways.^27^ Additionally, IL‐1α plays a role in angiogenesis by triggering the JNK signaling pathways and promoting VEGF expression.^28^ On the other hand, IL‐4, a significant anti‐inflammatory cytokine, promotes the differentiation of monocytes into M2 macrophages through its receptor,^29^ IL‐4R. In diabetic wounds, there is an abnormal, persistent polarization of M1 (pro‐inflammatory) macrophages,^30^ in contrast to normal wounds which typically transition towards M2 (pro‐healing) macrophages around the third day post‐injury.^31^ At DFU sites, macrophages often exhibit M1‐type polarization, and an increased presence and proportion of M1 macrophages can complicate the healing process.^32^ Given IL‐4's anti‐inflammatory effects and its role in monocyte differentiation,^33^ it could be pivotal in the wound repair response.

The study underscores the critical role of cytokines, especially IL‐6, in aiding vascular reconstruction in TTT technology. Further research is essential to fully understand the molecular mechanisms driving cytokine‐induced angiogenesis.

### Limitations of the Study

In this study, uniformity was maintained by having the same surgeon perform all surgeries using a consistent method, effectively minimizing biases associated with varying surgical skills and non‐standardized procedures. However, this study still has several limitations. Firstly, the limited size of the sample could potentially introduce bias in the outcomes. Upcoming research will be conducted on a larger scale and involve multiple centers. Secondly, in the subgroup analysis, patients with a history of hypertension had longer wound healing times compared with those without hypertension. This may be due to the smaller number of patients with preoperative hypertension, resulting in greater statistical bias. Third, this study lacked a control group. Thus, it did not compare the clinical outcomes of TTT with other conventional surgical treatments, such as vascular reconstruction. Consequently, there could be additional factors that complicate the interpretation of the findings. However, a previous multicenter study made such a comparison, finding that TTT had significantly higher ulcer healing and limb salvage rates, with a lower recurrence rate, compared with conventional surgical treatments.^7^ Fourth, in the process of using ABI to assess lower limb blood supply, there are several limitations due to the lack of a handheld 5–10 MHz Doppler ultrasound device: (1) Measurement Accuracy: The absence of a Doppler ultrasound device may lead to difficulties in identifying the pulse points of the ankle and brachial arteries, especially in patients with arterial sclerosis or circulatory disorders. This can affect the accuracy of measurements, particularly when pulse points are not discernible or difficult to palpate. (2) Assessment of Blood Flow: Doppler ultrasound devices are capable of detecting arterial blood flow velocity and direction, which is crucial for assessing the extent of vascular lesions and blood flow status. The lack of Doppler ultrasound may diminish the ability to comprehensively assess blood flow status, especially reducing sensitivity in cases of arterial stenosis or occlusion.

**FIGURE 5:**
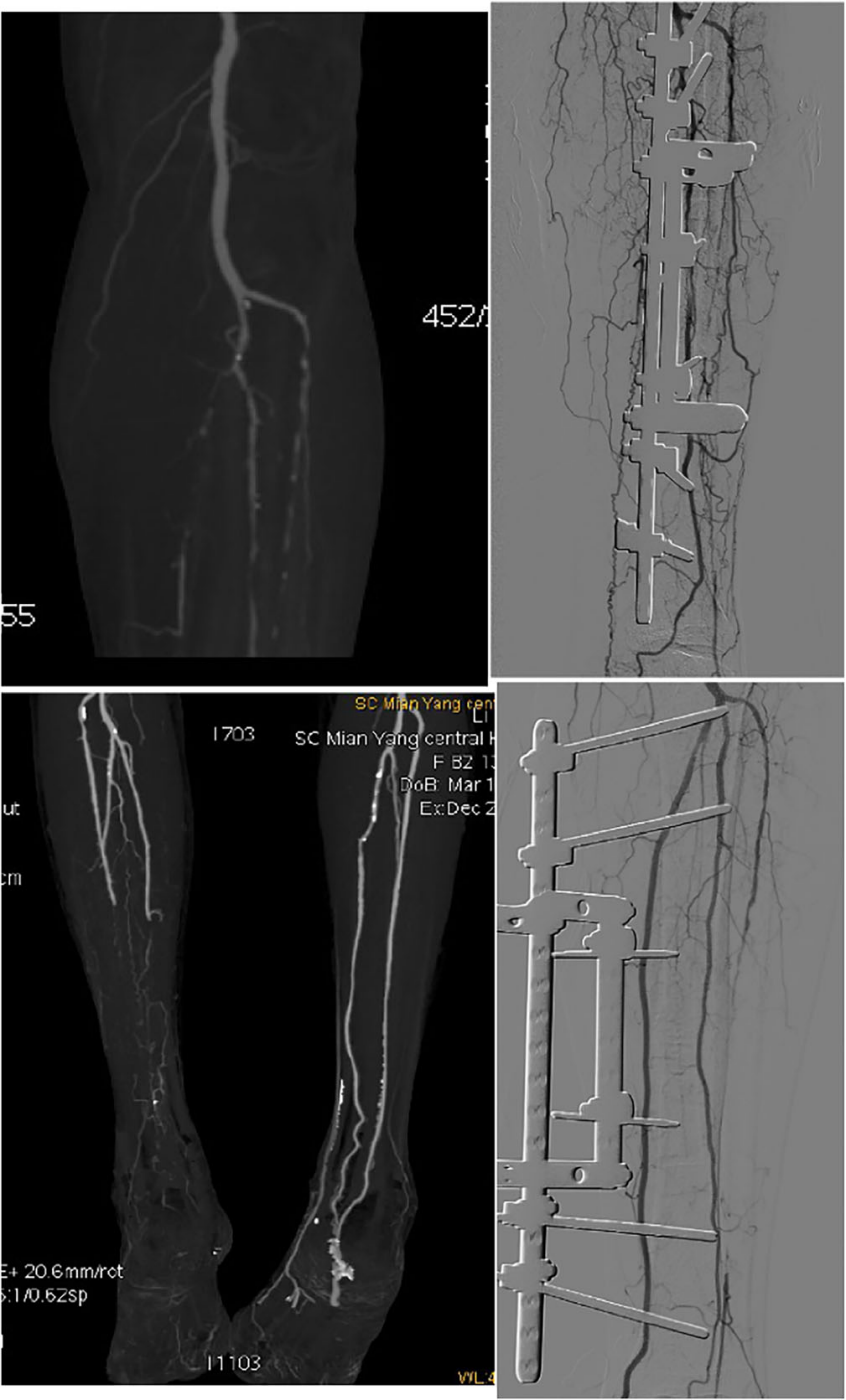
Arterial perfusion of the affected limb before and after tibial transverse translation (TTT).

In earlier clinical work, researchers observed enhanced vascular regeneration in patients undergoing TTT surgery through lower limb arterial perfusion, confirming its role in promoting angiogenesis (refer to Figure 5). Despite the aforementioned limitations, the ratio of ankle systolic blood pressure to brachial systolic blood pressure can still reflect lower limb perfusion. Here are some reasonable considerations and measures: (1) Basic Measurement Techniques: Even without a handheld 5–10 MHz Doppler device, accurate identification of ankle and brachial artery pulsation points can still be achieved through strict measurement methods and the training of experienced operators. (2) Repeated Measurements and Averaging: Conducting multiple measurements according to established procedures and averaging them can reduce the impact of measurement errors. This approach enhances the stability and reliability of the measurement results. (3) Clinical History and Observation: Integrating patients' clinical history and observational findings such as symptoms, signs, and other examination indicators can provide a more comprehensive assessment of lower limb perfusion. (4) Monitoring Treatment Effects: Despite potential limitations in measurement methods, ABI as a quantitative indicator can still be used to monitor treatment effects and improvements in vascular function. Therefore, despite technical and equipment constraints, accurate execution and interpretation of ABI measurements can still provide valuable clinical information.

In conclusion, we found that TTT technique surgeries are minimally invasive, resulting in shorter postoperative recovery periods and lower rates of complications compared with traditional methods. Cellular factors such as IL‐6 exhibit significant changes postoperatively compared with preoperative levels, suggesting potential involvement in vascular reconstruction. The role and mechanisms of IL‐6 require further confirmation through subsequent animal experimental research.

## Conclusion

The TTT technique can effectively increase the number of capillaries in the affected limb, improve blood supply, and promote wound healing. It has the ability to effectively and safely treat DFUs. However, it should be emphasized that the wound healing time in patients who smoke or consume alcohol is statistically significant compared with that of nonsmoking and nondrinking patients, stressing the need for tailored treatment and management approaches for effective therapy. L‐6 exhibited significant changes at various postoperative time points. Approaches that focus on modulating cytokines show the potential to accelerate the healing process of DFUs. To substantiate these findings, additional research involving a larger sample size is essential. Such research will provide valuable scientific data and lay a theoretical groundwork for subsequent research into the molecular mechanisms by which cytokines aid in vascular reconstruction.

## Funding Information

This study was supported by Special scientific research project on wound diseases (Taige) of Sichuan Medical Association Wound Disease (Taige) Special Research Project (2022TG26).

## Author Contributions

Xiaoya Li and Rong Hu were committed to conducting literature research. Fudie Guo participated in the clinical trials. Daofei Xu and Chunxia Bai performed data analysis and authored the manuscript. Dingwei Zhang and Bo Shi were responsible for the design of the study and made revisions to the manuscript. All the writers examined the manuscript prior to its submission.

## Conflict of Interest Statement

The authors declare no conflicts of interest.

## Ethics Statement

This study was approved by the institutional review board of Mianyang Central Hospital (S20230225‐01). Informed consent was obtained from all participants. Data were anonymized and securely stored. No conflicts of interest were reported. Funding was provided by Sichuan Medical Association Wound Disease (Taige) Special Research Project (No. 2022TG26).
